# Pharyngitis Workup Leads to the Discovery of Massive Tricuspid Vegetation

**DOI:** 10.7759/cureus.13375

**Published:** 2021-02-16

**Authors:** Matthew M Barvo, Jacob Pletz, Gbemisola Johnson, Muhammad Ayyaz

**Affiliations:** 1 Medicine, Trinity School of Medicine, Roswell, USA; 2 Internal Medicine, Coliseum Medical Centers, Macon, USA

**Keywords:** valvular endocarditis, tricuspid valve endocarditis, cardiology, tricuspid vegetation, pharyngitis

## Abstract

Infective endocarditis (IE) is one of the leading causes of life-threatening infections and is most often observed among patients who use intravenous (IV) drugs. We discuss the unique presentation of a 31-year-old gentleman with a two-week history of sore throat and shortness of breath, who returned to his community emergency room with persistent symptoms of streptococcal pharyngitis. A thorough history, physical examination, and diagnostic workup were conducted, where a large, protruding, highly mobile vegetation was observed on echocardiogram. His blood cultures grew methicillin-resistant *Staphylococcus aureus*. A vegetation measuring over 5 cm was surgically removed from his tricuspid valve. Following the operation, he underwent six weeks of extensive in-patient medical management with IV antibiotics to treat IE. This patient made a complete recovery and has since returned home.

## Introduction

Infective endocarditis (IE) is one of the top five most common causes of life-threatening infection syndromes, with a mortality rate of up to 25% [1]. This disease can attack different locations within the heart, including the right and left side of the heart, or it may infect an electronic cardiac device, or even a prosthetic valve [1]. Approximately 5-10% of IE cases are right-sided, and the tricuspid valve is the most common site for right-sided lesions. IE is commonly found in patients with intravenous (IV) drug abuse [2]. The most common pathogen for right-sided IE is *Staphylococcus aureus*, but other bacterial causes include the “HACEK” species, *Enterococci*, *Lactobacilli*, coagulase-negative *Streptococci *and *Staphylococci*, and even fungi such as *Candida *and *Aspergillus* [2]. Management of IE can include medical interventions, surgical interventions, or a combination of both. Medical management is successful in a majority of modern-day patients. When IE is clinically suspected, a provider should not wait for blood cultures. Instead, immediate therapy should be initiated with broad-spectrum antibiotics which cover the most common pathogens until blood cultures return [3]. Antibiotic therapy should be continued for up to six weeks to eradicate the infection [2,3]. Approximately 25% of IE cases require surgical management. Surgical management is usually required when a patient’s condition worsens to the point of congestive heart failure, in cases where the pathogen is resistant to antibiotics for over two weeks, in patients with septic pulmonary emboli, or when a vegetation exceeds 10 mm in size [2,3].

## Case presentation

This case reviews the medical and surgical management of a 31-year-old gentleman who presented to the emergency room (ER) with sore throat of two-week duration. He last visited the ER five days prior to the current admission and was diagnosed with streptococcal pharyngitis and discharged with antibiotics.

Physical examination in the ER revealed an ill-appearing man with a temperature of 103°F and scleral icterus. He was tachycardic with normal S1, S2 on auscultation. His lungs were without crackles, wheezes, or rhonchi. The abdomen was diffusely tender to palpitation with normal bowel sounds. Examination of his skin showed tract marks in antecubital fossa bilaterally and a Janeway lesion on the plantar surface of the right fourth toe.

Differential diagnoses in the ER included coronavirus disease 2019 (COVID-19), sepsis, community-acquired pneumonia, and IE. Rapid antigen for COVID-19 and blood cultures were drawn.

Laboratory examination revealed leukocytosis of 18.3 × 10^3^/µL, elevated lactic acid of 2.3 mmol/L, elevated total bilirubin of 3.3 mg/dL, and elevated liver enzymes with aspartate transaminase of 237 U/L, alanine transaminase of 310 U/L, and alkaline phosphatase of 239 U/L. Urine toxicology was positive for opiates, amphetamine, and benzodiazepines. Blood cultures grew methicillin-resistant *Staphylococcus aureus* (MSSA). The test for COVID-19 was negative.

Chest X-ray revealed a normal cardiac silhouette, bibasilar atelectasis, and multifocal ground glass infiltrates bilaterally. Computed tomography arteriogram revealed patchy and cavitary pulmonary lesions suggestive of septic emboli, with filling defects within the segmental and sub-segmental pulmonary arteries, which were concerning for septic emboli. Multi-loculated pleural effusions were noticed bilaterally, representing empyemas.

**Figure 1:**
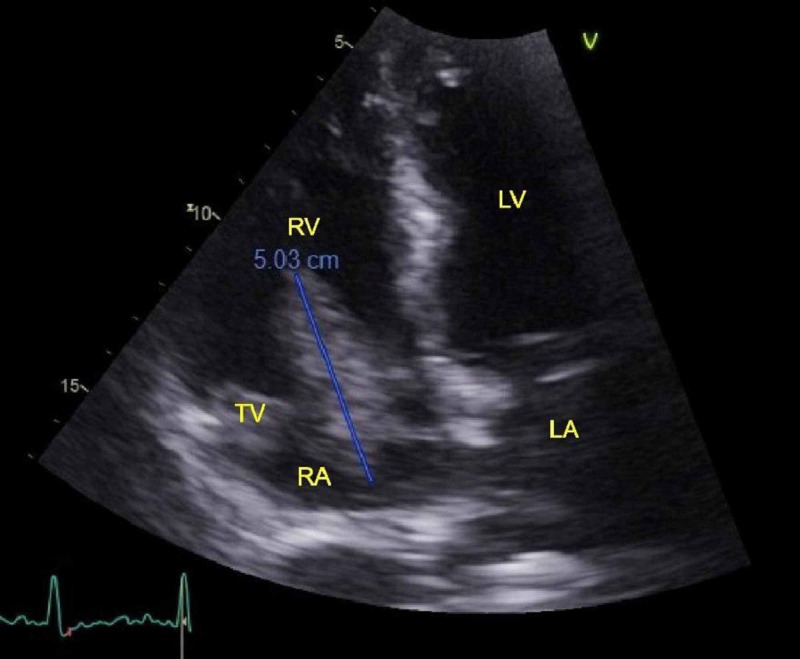
Highly mobile 5 cm vegetation on the tricuspid valve. TV, tricuspid valve; RV, right ventricle; LV, left ventricle; RA, right atrium; LA, left atrium

The patient was transferred from the ER to the affiliated tertiary care center for medical and surgical management. Repeat blood cultures grew MSSA and he was started on IV nafcillin, vancomycin, and high-dose heparin drip. Transesophageal echocardiogram showed a large, protruding, echogenic, and highly mobile vegetation on the tricuspid valve measuring over 5 cm (Figure 1). Cardiothoracic surgery was consulted. Infectious disease specialist started nafcillin drip.

Cardiothoracic surgery team excised a 5 cm vegetation that was adhered to the anterior and posterior leaflets of the tricuspid valve. The valve leaflets were repaired with a pericardial patch and an incomplete David ring was placed along the tricuspid annulus. The bilateral empyemas were drained. He was returned to the intensive care unit where he was extubated two days later. He tolerated the surgery well without major complications in the postoperative period.

**Figure 2:**
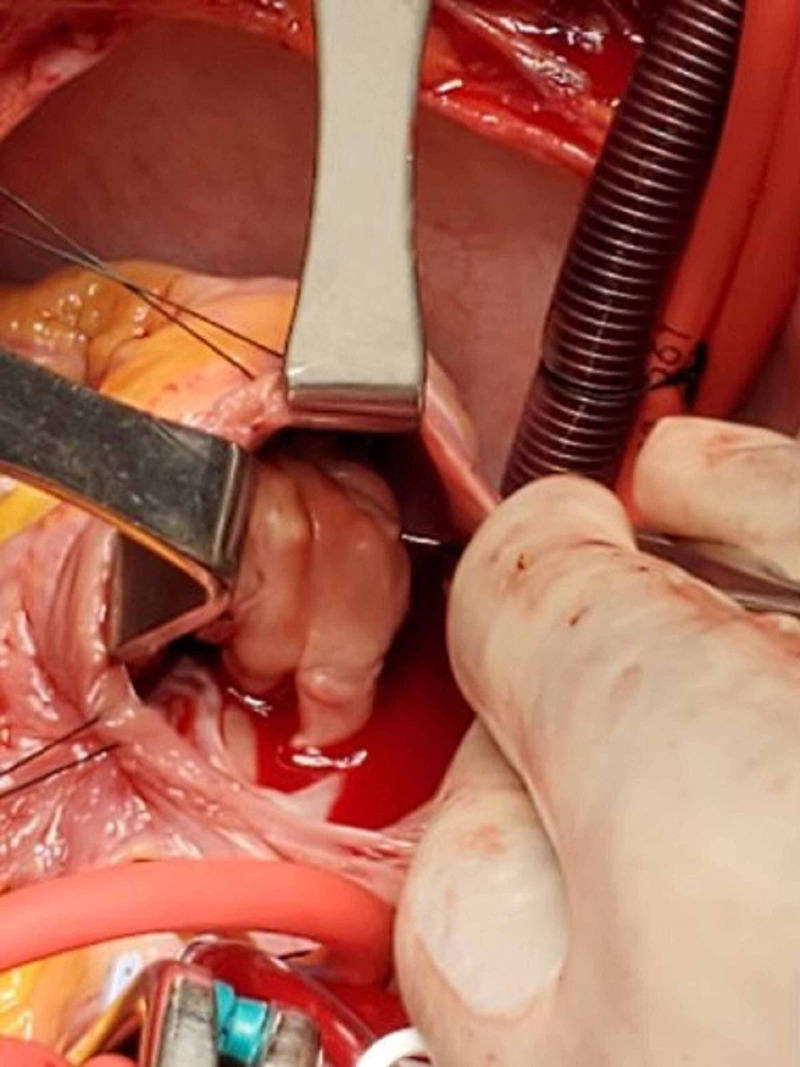
Looking into the right atrium to observe the 5 cm vegetation during the surgery.

The patient remained in the hospital for six weeks to receive the IV nafcillin regimen. As he lacked health insurance, had a history of illicit IV drug use, and had a vascular access port to receive antibiotics, he could not be discharged home. The patient was eventually transferred to a long-term rehabilitation center for antibiotic and pain management administration, and has since made a complete recovery.

## Discussion

Only 5-10% of IE cases affect the right side of the heart [2]. Drug usage is known to be a leading cause of endocarditis, and up to 41% of drug users presenting to the hospital with a fever are found to have echocardiographic evidence of endocarditis [4]. Although there are many potential causative agents, *S. aureus* is most commonly associated with IV drug abuse and accounts for 70% of all IE cases [2,4].

How would a clinician differentiate the symptoms of IE from streptococcal pharyngitis? The initial diagnostic decision would begin with a thorough history, which may illustrate a predisposing heart condition such as mitral valve prolapse or bicuspid aortic valve, or the patient may confide a history of illicit IV drug use. During the physical examination, a clinician would need to carefully differentiate Janeway lesions from the lesions of astreptococcal rash. These findings discovered during the history and physical examination would then be evaluated using the Duke Criteria, instead of just being attributed to the sequelae of strep throat.

The Duke Criteria is a diagnostic criteria which helps evaluate patients with suspected IE [5]. This criteria has a sensitivity of 76% for diagnosing IE and comprises pathological, major, and minor clinical criteria [6]. Upon presenting to the ER, our patient satisfied three of the five minor clinical criteria, as well as history of IV drug abuse, fever, and Janeway lesion on the foot. This patient eventually satisfied both major criteria.

IE can successfully be managed with medical and surgical treatments to achieve the best overall prognosis, as seen with the patient in this case. Patients who are treated medically in a hospital setting have a 12% mortality rate [7]. Medical management should utilize broad-spectrum antibiotics and continue for a duration of six weeks [3]. The antibiotic regimen should be started immediately in anticipation of the suspected pathogens that are the routine causative agents. Surgical patients also have excellent outcomes, with mortality rates less than 7.3% [8]. Surgical management becomes the treatment of choice when medical management fails or is prolonged beyond two weeks without improvement [3]. When a valvular vegetation is found to be larger than 10 mm, as was observed in our patient, surgical intervention should be strongly considered. Furthermore, as pulmonary embolism is a clinical sign of IE, as seen in our patient, anticoagulation therapy is important to minimize complications [8].

## Conclusions

Patients who present with symptoms similar to this patient should be evaluated with care to differentiate the signs of IE from the various sequelae of streptococcal infection. This way, a potential IE will not be overlooked, can be investigated further, and treated sooner, resulting in a significantly reduced mortality rate.
